# Obstructive sleep apnea and cardiac arrhythmias

**DOI:** 10.4103/1817-1737.58954

**Published:** 2010

**Authors:** Ahmad Salah Hersi

**Affiliations:** *King Fahad Cardiac Centre, College of Medicine, King Saud University, Riyadh 11472, Saudi Arabia*

**Keywords:** Arrhythmia, OSA, sleep apnea

## Abstract

Sleep-disordered breathing (SDB), which includes obstructive sleep apnea (OSA) as its most extreme variant, is characterized by intermittent episodes of partial or complete obstruction of the upper airway, leading to cessation of breathing while asleep. Cardiac arrhythmias are common problems in OSA patients, although the true prevalence and clinical relevance of cardiac arrhythmias remains to be determined. The presence and complexity of tachyarrhythmias and bradyarrhythmias may influence morbidity, mortality and quality of life for patients with OSA. Although the exact mechanisms underlying the link between OSA and cardiac arrhythmias are not well established, they could be some of the same proposed mechanisms relating OSA to different cardiovascular diseases, such as repetitive pharyngeal collapse during sleep, which leads to markedly reduced or absent airflow, followed by oxyhemoglobin desaturation, persistent inspiratory efforts against an occluded airway and termination by arousal from sleep. These mechanisms elicit a variety of autonomic, hemodynamic, humoral and neuroendocrine responses that evoke acute and chronic changes in cardiovascular function. However, despite substantial research effort, the goals of determining in advance which patients will respond most favorably to certain treatment options (such as continuous positive airway pressure, tracheostomy or cardioversion) and the developing alternative treatments remain largely elusive. Therefore, this literature review aims to summarize a broad array of the pathophysiological mechanisms underlying the relationship between OSA and cardiac arrhythmias and the extent of this association from an epidemiological perspective, thereby attempting to assess the effects of OSA treatment on the presence of cardiac arrhythmias.

Sleep-disordered breathing (SDB) describes a group of disorders characterized by abnormalities in the frequency and/or depth of breathing while asleep. The major risk factors for SDB include obesity, male gender, increasing age and abnormalities of craniofacial morphology.[1,2] The different types of SDB include obstructive sleep apnea (OSA)/hypopnea syndrome (OSAHS), obesity hypoventilation syndrome, central sleep apnea, upper-airway resistance syndrome and Cheyne–Stokes respiration.[3,4] Obstructive sleep apnea syndrome (OSAS), the most extreme variant of SDB, is characterized by intermittent episodes of partial or complete obstruction of the upper airway during sleep, which disrupts normal ventilation and sleep architecture and is typically associated with snoring and daytime sleepiness.[5–8] It affects 4% of men and 2% of women aged 30-65 years, making it at least as common as type I diabetes.[9] A diagnosis of OSAS is accepted when a patient has an apnea–hypopnea index (AHI; number of apneas and hypopneas per hour of sleep) >5 and symptoms of excessive daytime sleepiness based on polysomnographic examinations.[1]

Over the last decade, the association between OSA and cardiac rhythm disorders has garnered the attention of researchers from different clinical subspecialties[10–12] since cyclic variation in heart rate is considered typical in SDB. Cardiac arrhythmias are presumed to be a common problem in patients with OSA, although the true prevalence and clinical relevance of cardiac arrhythmias remain unknown. The presence and complexity of tachyarrhythmias and bradyarrhythmias may influence morbidity, mortality and the quality of life for OSA patients[13,14] Although the exact mechanisms underlying the link between OSA and cardiac arrhythmias are not well established, they could be some of the same proposed mechanisms relating OSA to different cardiovascular diseases. OSA leads to a repetitive pharyngeal collapse during sleep, followed by oxyhemoglobin desaturation, persistent inspiratory efforts against an occluded airway, and termination by arousal from sleep.[15] These mechanisms elicit a variety of autonomic, hemodynamic, humoral and neuroendocrine responses that by themselves evoke acute and chronic changes in cardiovascular function. These effects may lead to the development of cardiac arrhythmias or other form of cardiovascular diseases linked to OSA.[16–18] The most common arrhythmias during sleep include nonsustained ventricular tachycardia, sinus arrest, second degree atrioventricular conduction block and frequent (>2 bpm) premature ventricular contractions.[19–23]

The incidence and prevalence of arrhythmias in the setting of SDB are poorly defined due to smaller number of studies and inadequate control for potential confounders.[24–27] Despite the biological plausibility for OSA-associated hypoxemia, arousals and autonomic nervous system dysregulation to cause generation of abnormal cardiac electrophysiologic impulses, few studies have rigorously characterized the association between OSA and cardiac arrhythmias. Therefore, controversy still remains as to whether OSA is a primary etiologic factor for tachyarrhythmias, due to high incidence of cardiovascular comorbidities in patients diagnosed with OSA. The prognostic significance of arrhythmias occurring during OSA is also unknown. It is still not clear whether treatment of OSA or arrhythmias has a significant impact on cardiovascular morbidity and mortality.

This review of literature summarizes a broad array of the pathophysiological mechanisms underlying the relationship between OSA and cardiac arrhythmias and the extent of this association from an epidemiological perspective, thereby attempting to assess the effects of OSA treatment on the prevalence of cardiac arrhythmias.

## Epidemiology

OSAS is a common breathing disorder, affecting approximately 2%-4% of total population, the prevalence in men being almost twice that of women.[28–30] The prevalence of undiagnosed OSAS is up to 5% for adults in Western countries.[31] There are many different types of arrhythmias linked to OSAS. Cardiac arrhythmias such as atrial and ventricular premature extrasystoles, nonsustained ventricular tachycardia, sinus arrest and second-degree atrioventricular conduction block are reportedly 30%-50% in patients with OSA and increase with the number of apneic episodes and severity of the associated hypoxemia.[32–34] Other tachyarrhythmias, such as persistent supraventricular tachycardia, atrial fibrillation or flutter, and ventricular arrhythmias, particularly sustained or nonsustained ventricular tachycardia are more likely to occur in the setting of pre-existing structural heart disease.[35] Initial isolated case reports hinted at an association between OSA and bradyarrhythmias.[36] The most common arrhythmias during sleep including nocturnal arrhythmias have been shown to occur in up to 50% of OSA patients. The prevalence of atrial fibrillation (AF) is 0.4% in the general population and >6% for population over 80 years.[37] On the basis of an obstructive apnea frequency, in middle age, 24% of men and 9% of women have OSA.[38] However, there is a wide discrepancy in the reported prevalence of all types of cardiac arrhythmias in OSA.[39,40] Moreover, conclusions as to whether arrhythmias are more common among those with SDB are conflicting.[39,40] Table 1 summarizes various studies relating prevalence of OSA and cardiac arrhythmias.

**Table 1:** Prevalence studies of cardiac arrhythmias and obstructive sleep apnea

Studies	Subjects	Outcomes/Prevalence
Tilkian *et al*.[45]	15	Marked sinus arrhythmia in14 patients
		Extreme sinus bradycardia in 6
		Asystole in 5
		Second-degree atrioventricular block in 2
		Ventricular arrhythmias--complex premature ventricular beats in 10
		Ventricular tachycardia in 2 patients
Guilleminault *et al*.[22]	400	Bradyarrhythmias in 18% of patients
		Sustained ventricular tachycardia in 2%
		Sinus arrest in 11%
		Second-degree atrioventricular block in 8%
		Frequent premature ventricular contractions in 19%
Flemons *et al*.[39]	263	Complex ventricular ectopy (including ventricular tachycardia) in 1.3% of patients
		Frequent ventricular premature beats (>30/h) in 2.6%
		Second-degree atrioventricular block in 1.3%
		Sinus arrest in 5.2% patients
Becker *et al*.[46]	239	Sinus arrest and atrioventricular (AV) block in 30% of patients
Mooe *et al*.[47]	121	Atrial fibrillation (AF) in 32% of patient with apnea–hypopnea index (AHI) >5 or =5 and in 18% patients with AHI <5
		Atrial fibrillation in 39% of patients with oxygen desaturation index (ODI) >5 or =5 and in 18% of patients with ODI <5
Javaheri *et al*.[48]	81	Atrial fibrillation in 32% of patients
Simantirakis *et al*.[50]	23	Rhythm disturbances in 48% of patients
Gami *et al*.[51]	524	OSA more prevalent in patients with AF (n = 151) than in high-risk patients with multiple other cardiovascular diseases
Porthan *et al*.[52]	115	Sleep apnea syndrome common in lone AF
Mehra *et al*.[18]	566	Atrial fibrillation in 4.8% of patients
		Nonsustained ventricular tachycardia in 5.3%
		Complex ventricular ectopy in 25.0% of patients

From various data, it appears that arrhythmias were more common in SDB patients who have severe nocturnal hypoxemia during rapid eye movement sleep.[40] A recent study among patients who had undergone successful cardioversion for atrial fibrillation demonstrated an 82% rate of recurrence over a 12-month period in contrast to 42% and 53% rates of recurrence among OSA patients treated with continuous positive airway pressure (CPAP) and controls who did not undergo a sleep study, respectively.[41] The role of OSA as an independent risk factor for cardiac arrhythmias, particularly for life-threatening arrhythmias is still unresolved. Successful treatment for OSA with CPAP seems to decrease arrhythmias.[42] However, no study has addressed the independent role of OSA on arrhythmic death as opposed to the confounding effect of coexisting diabetes and cardiovascular disease.[43] For the relationship between OSA and arrhythmias to be clarified, carefully designed prospective studies are necessary.

## OSA in Children

In children, OSAS is characterized by central hypoventilation and disorders of respiratory muscles. Children with comorbid conditions, such as genetic syndromes that may alter airway anatomy, central control of breathing, or respiratory muscle function represent a large percentage of pediatric patients presenting to pediatric sleep centers with OSA. OSA in children is frequently due to adenotonsillar hypertrophy with airway narrowing, with only a modest role for obesity. However, there is scarcity of literature pertaining to the estimation of cardiovascular risk from OSA in children.[44]

## Evidences Linking Sleep Apnea with Cardiac Arrhythmias

The association between OSAS and arrhythmias was first documented over 30 years ago. In a small study conducted by Tilkian *et al.*, the effect of atropine and tracheostomy on cardiac arrhythmias during wakefulness and sleep in 15 patients with sleep-induced obstructive apnea were studied by continuous overnight Holter electrocardiographic, respiratory and electroencephalographic recordings.[45] Sleep was associated with marked sinus arrhythmia (93%), extreme sinus bradycardia (40%), asystole (33%), second degree atrioventricular (A-V) block (13%), ventricular arrhythmias--complex premature ventricular beats (66%) and ventricular tachycardia (13%). Arrhythmias during wakefulness were limited to premature ventricular beats (40%). The results showed marked sinus arrhythmia during sleep as a characteristic of OSA, frequently accompanied by potentially life-threatening tachyarrhythmia and bradyarrhythmia.

Guilleminault *et al.* studied 400 patients with OSA; of these, 48% had documented cardiac arrhythmias, including bradyarrhythmias in 18%, sustained ventricular tachycardia in 2%, sinus arrest in 11%, second-degree atrioventricular block in 8% and frequent premature ventricular contractions in 19%.[22] The mean number of apneic events, age, weight and lowest oxygen saturation during sleep were not significantly different in those with arrhythmias as compared with those with conduction disturbances. The most significant abnormalities were unsustained ventricular tachycardia in 8 patients, sinus arrest that lasted for 2.5-13 seconds in 43 patients, and second-degree atrioventricular conduction block in 31 patients. Seventy-five patients had frequent (>2 beats/min) premature ventricular contractions during sleep. Fifty patients with significant arrhythmias had a tracheostomy and were monitored again after surgery. However, no arrhythmias, except for premature ventricular contractions, were present in these patients after surgery.

In contrast to the above findings, Flemons *et al.* observed the prevalence of arrhythmias in 263 physician-referred patients in determining a relationship between cardiac arrhythmias and sleep apnea.[39] The prevalence of arrhythmias in patients with and without sleep apnea was: complex ventricular ectopy (including ventricular tachycardia)-1.3% versus 4.1%; frequent ventricular premature beats (> 30/h)-2.6% versus 6.2%; second-degree atrioventricular block-1.3% versus 4.1% and sinus arrest- 5.2% versus 1.0%. The differences were statistically insignificant and the presence or absence of arrhythmias seemed to be unrelated to sleep apnea severity.

To explore these seemingly discrepant results, Becker *et al.* performed Holter monitoring in 239 consecutive patients diagnosed with sleep apnea using a validated ambulatory recording device based on the measurement of heart rate, oxygen saturation (SaO_2_), snoring sound, and body position.[46] The observations indicated that bradycardic arrhythmias occurred exclusively during apneas and hypopneas and were not found during hyperventilation, thereby signifying a clear link between bradycardic arrhythmias and apnea severity.

The 1995 Becker study was followed by another observational study conducted by Mooe *et al.*, wherein they hypothesized that preoperatively diagnosed SDB with nocturnal hypoxemia was an independent predictor of atrial fibrillation after coronary bypass surgery.[47]

Javaheri *et al.* studied 81 ambulatory male patients with stable heart failure without major comorbid disorders. Of these, 51% suffered from sleep-related breathing disorder, of which 40% from central and 11% from OSA.[48] Patients with sleep apnea had a significantly higher prevalence of AF and ventricular arrhythmias. Similarly, patients with an AHI ≥5 undergoing coronary artery bypass surgery were found to have a higher incidence of postoperative AF (32% vs. 18% in patients without SDB).

Continuous cardiac monitoring with an atrial defibrillator showed that onset of nearly 75% of episodes of persistent atrial fibrillation occurred between 8 pm and 8 am, which could partially be explained by the presence of OSA.[49]

In an elegant study, Simantirakis *et al.* inserted loop recording devices in 23 patients with OSAHS in whom other cardiac and pulmonary diseases were first excluded through exercise tests, invasive electrophysiological studies, echocardiography and lung function tests; patients with diabetes were excluded.[50] Over 2 months of continuous recording, 48% had significant rhythm disturbances, mostly occurring at night. They also noted that 48-h Holter monitoring was not able to detect bradyarrhythmic events. Gami *et al.* prospectively studied consecutive patients undergoing electrocardioversion for AF (n = 151) and consecutive patients without past or current AF referred to a general cardiology practice (n = 312).[51] The presence of OSA was determined by the Berlin questionnaire, a validated questionnaire to identify individuals at high risk to OSA. The proportion of patients with OSA was significantly higher in the AF group than in the general cardiology group (49% vs. 32%). The novel finding of this study was that a strong association exists between OSA and AF, such that OSA was strikingly more prevalent in patients with AF than in high-risk patients with other multiple cardiovascular diseases. In contrast, a smaller Finnish study, using objective sleep studies (AutoSet Portable II Plus device), disagreed with the previous findings and actually found no difference in the prevalence of OSAHS in 59 patients with lone AF compared with 56 controls matched for age, gender and cardiovascular morbidity.[52]

A subgroup analysis of the Sleep Heart Health Study revealed a fourfold increase in the prevalence of AF in subjects with an AHI >30 as compared with patients with no SDB matched for age, sex, ethnicity and BMI.[18] A significant relation was also observed between SDB and the number ventricular ectopic per hour.

To summarize the above findings, it can be rightly stated that individuals with severe SDB were found to have two- to fourfold higher odds of complex arrhythmias than those without SDB.

## Pathophysiological Mechanisms of Arrhythmias in OSA

**Figure 1:**
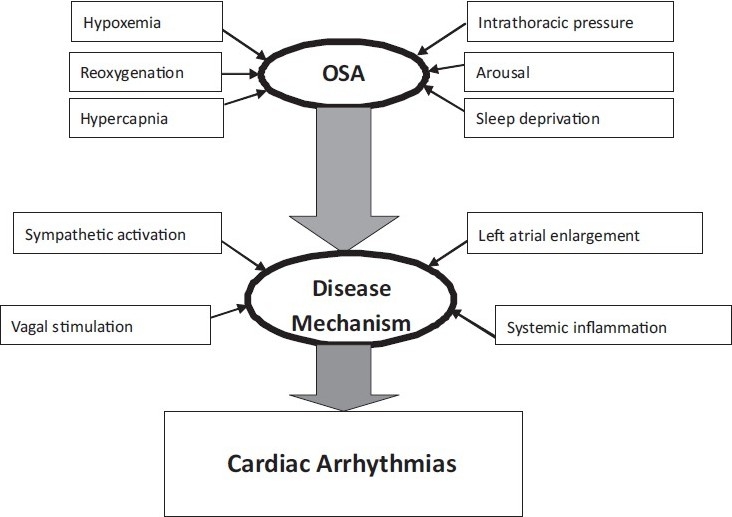
Schematic representation of various pathophysiological mechanisms relating to arrhythmias in obstructive sleep apnea (OSA)

Although an understanding of the exact mechanisms is not well established, there are several possible mechanisms by which arrhythmias would occur more commonly or be more severe in the presence of OSA [Figure 1].

OSA is responsible for repeated blood oxygen desaturations and concomitant increase in arterial carbon dioxide levels due to dysfunction in baroreflex[53,54] and chemoreflex,[55,56] which leads to activation of the sympathetic nervous system. A persistent increase in sympathetic tone has been shown to generate abnormal electrical remodeling of the atrium, thus facilitating supraventricular arrhythmias, particularly atrial fibrillation.[57] Electric remodeling may create some degree of interatrial block, contributing to the genesis of atrial arrhythmias.[58] Enhanced sympathetic nervous system activity associated with respiratory event-related hypoxemia and arousal may trigger automaticity due to a stimulated action potential.[59]

A strong association between OSA and hypertension has been extensively reported[60,61] and the association between hypertension and AF is also well recognized.[62,63] Although purely speculative, the link between OSA and AF could merely be the distortion of the atrial anatomy that occurs during hypertension.[64] The forceful ventilatory efforts against upper airway obstruction during apneas result in dramatic shifts in transmural pressures and measurable changes in cardiac chamber dimensions.[65,66] These acute structural changes may promote AF by triggering stretch-activated atrial ion channels.[67] The severity of OSA is independently associated with elevated markers of systemic inflammation, including C-reactive protein[68] which is directly associated with an increased AF burden.[69] Although sleep apnea has been associated with left atrial enlargement,[70,71] the suggestion that it leads to atrial fibrillation, is an appealing but presently unproven hypothesis.

In contrast, bradyarrhythmias are probably related to the prolonged apnea and hypoxemia in OSA that elicit the cardiac vagal activation reflex, with simultaneous sympathetic activation to the peripheral blood vessels, including muscle, renal and splanchnic but not cerebral vasculature.[72–75] Although the vagal response will often elicit a discernible bradycardia, in a minority of OSA patients (approx. 10%), bradyarrhythmias such as atrioventricular block and asystole may develop even in the absence of cardiac conduction disease.[22] These are most likely to occur during rapid eye movement sleep and with a decrease in oxygen saturation of at least 4%.[76] Re-entry mechanisms may occur through the vagal stimulation that results from respiration against a partially occluded airway, which may lead to bradycardia-dependent increased dispersion of atrial repolarization predisposed to intra-atrial entry.[77,78]

Additionally, SDB-related mechanical effects of negative intrathoracic pressure on the atrial and ventricular free walls promote cardiac stretching, which may predispose one to arrhythmias by way of mechanical electrical feedback mechanisms.[79]

## Treatment

### Treatment of sleep apnea with respect to arrhythmias

There are no conclusive epidemiologic or longitudinal intervention studies that relate specifically to the prevalence, severity and consequences of cardiac arrhythmias and effects of OSA treatment. If the underlying cardiac conduction system is normal and there is no significant arrhythmia or heart block occurring during apneic periods, it may be treated effectively with CPAP or, if necessary, with tracheostomy.[53,80]

The 1995 study of Becker *et al.*,[54] revealed that 7% of 239 (17 patients) with OSAHS had significant bradyarrhythmias and of these 17 patients, only 1 continued to experience bradyarrhythmias after CPAP therapy. Most of these patients had established or newly diagnosed cardiac abnormalities (hypertension, congestive cardiac failure, pulmonary hypertension); hence, an independent effect of OSAHS was difficult to establish.

Observational data put forward by Kanagala *et al.* showed an increased rate of recurrence (82%) of AF after successful cardioversion in inadequately treated patients with OSAHS as compared with non-OSAHS and well-treated OSAHS patients.[41]

Harbison *et al.* investigated the prevalence of significant cardiac rhythm disturbance in 45 patients with established moderate to severe OSAS and assessed the impact of nasal CPAP therapy.[42] The results showed an abolition of rhythm disturbances in seven of the eight patients, who had pathologically significant disturbances such as ventricular tachycardia or fibrillation, complex ventricular ectopy or new-onset supraventricular tachycardia.

In a randomized controlled 1-month trial involving 18 patients with OSA and systolic dysfunction, Ryan *et al.* tested the effects of CPAP therapy on ectopic ventricular arrhythmias.[81] The results suggested that in patients with heart failure (HF), treatment of coexisting OSA by CPAP reduces the frequency of VPBs by 58% during sleep, which might improve the prognosis in patients with HF patients.

In the longest-term prospective cohort study yet published (10 y), Marin *et al.* demonstrated a higher risk of both fatal and nonfatal cardiovascular events in men with severe OSA who were noncompliant with CPAP therapy as compared with snorers, CPAP-treated patients with OSA and healthy men.[82] Although biased by potential and difficult-to-measure influences related to treatment noncompliance and imbalances in some confounding variables at baseline (such as prevalence of hypertension and glucose intolerance) this study is among the most persuasive to argue that OSA has detrimental effects on long-term cardiovascular outcomes.

However, any therapeutic strategy that would incorporate long-term atrial pacing would need to recognize the potential interactions between OSA and atrial fibrillation. In patients cardioverted for atrial fibrillation, the presence of untreated sleep apnea doubles the likelihood of recurrence of atrial fibrillation within 12 months as compared with OSA patients receiving CPAP therapy.[41]

### Cardiac pacing as a treatment of sleep apnea

An initial report has, however, added new perspective to pacemaker placement in patients with OSA.[83] Interactions between cardiac arrhythmias and OSA may extend beyond the traditional concepts of OSA as a cause of abnormalities in cardiac rhythm. Provocative evidence suggests that modulation of cardiac rhythm characteristics by atrial overdrive pacing may attenuate the severity of both OSA and central sleep apnea. The mechanisms of any pacing-induced amelioration of sleep apnea and the implication for future therapeutic strategies are presently uncertain, but intriguing.

However, these finding were not replicated in subsequent investigations involving somewhat different patient populations; 16 patients with the OSA were prospectively evaluated for the effect of atrial overdrive pacing after 24 hours and after 1 month compared with the use of nasal CPAP.[84–86] The outcome highlighted that atrial overdrive pacing had no significant effect on OSA severity, whereas CPAP was highly effective in treating OSA.

A prospective, single-blinded, randomized crossover trial in patients with moderate to severe OSA also showed that temporary atrial pacing does not appear to improve respiratory manifestations of OSA. Permanent atrial pacing in this patient population does not appear to be justified.[87]

Recent data confirm that overdrive pacing exerts a mild effect on respiratory events in some heart failure patients with OSA. However, atrial overdrive pacing was not therapeutically effective for improving airway patency and sleep-related respiratory function.[88] Thus, there is currently no definitive evidence to support atrial overdrive pacing as a treatment option for OSA.

## Future Directions

If the presence of arrhythmias influencing SDB prognosis is uncertain; it speculates a rationale for systematic arrhythmia detection and reporting in the sleep laboratory.[89] The various challenges that need to be addressed include widespread comorbidities, including obesity, that obscure clearer understanding of any independent cardiovascular consequence of sleep apnea per se, treatment options that are varied, predominantly device based and not easily tolerated and the absence of robust longitudinal interventional studies addressing whether the treatment of sleep apnea confers any tangible benefit in terms of arrhythmias. Moreover, there is no clear evidence as to the best measurement for quantifying sleep apnea severity.

Available prevalence studies and interventional trials suggest that SDB and OSA, in particular, may cause and exacerbate arrhythmias. The mechanism(s) behind these associations are biologically plausible and are still being elucidated. Awareness of the association of cardiovascular disease and OSA will prompt the general practitioners to look for both illnesses when presented with one of them. There is no doubt that CPAP and other treatments for OSA will make symptomatic patients who happen to have a cardiac disease feel better. It may be that CPAP will be used to improve cardiac outcomes and randomized controlled trials of CPAP are being designed in patients with cardiovascular risk and OSA who do not exhibit sleepiness.

To demonstrate a causative relationship between OSA and cardiac arrhythmias, further prospective trials demonstrating a reduction in the incidence or recurrence of AF with effective treatment of OSA are needed.[64,90] Understanding the mechanisms that link sleep apnea to cardiac arrhythmias is essential and its early recognition may contribute positively in reducing risk of patients. Appropriate referral to sleep disorder clinics may facilitate definitive diagnosis and treatment initiation necessary.

Further large-scale work is also needed to quantify the population attributable risk of atrial fibrillation and other arrhythmias associated with OSA, the prognostic implications of these arrhythmias and the ability to prevent or reverse these arrhythmias with economical and better-tolerated therapeutic options. These could thereby confirm whether therapy attenuates cardiovascular morbidity and mortality and defining appropriate therapeutic targets and cost-effective benefits of such therapy.

## Conclusion

The evidence reviewed in this paper emphasizes the association between SDB, in particular, OSA and cardiovascular problems, particularly arrhythmias, and provides some insight into the mechanisms potentially involved in determining this association. It also substantiates the idea that, despite significant research effort, the goal of determining in advance which patients will respond most favorably to certain treatment options such as CPAP, tracheostomy or cardioversion ahead of time and developing alternative treatments remain largely elusive.

## References

[1] Partinen M (1995). Epidemiology of obstructive sleep apnea syndrome. Curr Opin Pulm Med.

[2] Parati G, Lombardi C, Narkiewicz K (2007). Sleep apnea: Epidemiology, pathophysiology, and relation to cardiovascular risk. Am J Physiol Regul Integr Comp Physiol.

[3] American Academy of Sleep Medicine Task Force (1999). Sleep-related breathing disorders in adults: Recommendations for syndrome definition and measurement techniques in clinical research The Report of an American Academy of Sleep Medicine Task Force. Sleep.

[4] Malhotra A, White DP (2002). Obstructive sleep apnea. Lancet.

[5] Quan SF, Gersh BJ (2004). Cardiovascular consequences of sleep-disordered breathing: Past, present and future report of a workshop from the national center on sleep disorders research and the national heart, lung, and blood institute. Circulation.

[6] Lattimore JD, Celermajer DS, Wilcox I (2003). Obstructive sleep apnea and cardiovascular disease. J Am Coll Cardiol.

[7] Shamsuzzaman AS, Gersh BJ, Somers VK (2003). Obstructive sleep apnea: Implications for cardiac and vascular disease. JAMA.

[8] Bradley TD, Floras JS (2003). Sleep apnea and heart failure: Part I: Obstructive sleep apnea. Circulation.

[9] Young T, Palta M, Dempsey J, Skatrud J, Weber S, Badr S (1993). The occurrence of sleep-disordered breathing among middle-aged adults. N Engl J Med.

[10] Spitz A (1937). Das klinische syndrome:Narcolepsy mit Fettsucht und Polyglobulien seinem Beziehungen zum morbus Cushing. Deutsch Arch Klin Med.

[11] Wolk R, Kara T, Somers VK (2003). Sleep-disordered breathing and cardiovascular disease. Circulation.

[12] Pack AI (2006). Advances in sleep-disordered breathing. Am J Respir Crit Care Med.

[13] Narkiewicz K, van de Borne PJ, Pesek CA, Dyken ME, Montano N, Somers VK (1999). Selective potentiation of peripheral chemoreflex sensitivity in obstructive sleep apnea. Circulation.

[14] Dimsdale JE, Loredo JS, Profant J (2000). Effect of continuous positive airway pressure on blood pressure: A placebo trial. Hypertension.

[15] Narkiewicz K, van de Borne PJ, Cooley RL, Dyken ME, Somers VK (1998). Sympathetic activity in obese subjects with and without obstructive sleep apnea. Circulation.

[16] Arias MA, Sánchez AM (2007). Obstructive sleep apnea and its relationship to cardiac arrhythmias. J Cardiovasc Electrophysiol.

[17] Parish JM, Somers VK (2004). Obstructive sleep apnea and cardiovascular disease. Mayo Clin Proc.

[18] Mehra R, Benjamin EJ, Shahar E, Gottlieb DJ, Nawabit R, Kirchner HL (2006). Association of nocturnal arrhythmias with sleep-disordered breathing: The sleep heart health study. Am J Respir Crit Care Med.

[19] Koehler U, Schafer H (1996). Is obstructive sleep apnea (OSA) a risk factor for myocardial infarction and cardiac arrhythmias in patients with coronary heart disease (CHD)?. Sleep.

[20] Liston R, Deegan PC, McCreery C, McNicholas WT (1994). Role of respiratory sleep disorders in the pathogenesis of nocturnal angina and arrhythmias. Postgrad Med J.

[21] Shepard JW (1992). Hypertension, cardiac arrhythmias, myocardial infarction, and stroke in relation to obstructive sleep apnea. Clin Chest Med.

[22] Guilleminault C, Connolly SJ, Winkle RA (1983). Cardiac arrhythmia and conduction disturbances during sleep in 400 patients with sleep apnea syndrome. Am J Cardiol.

[23] Hoffstein V, Mateika S (1994). Cardiac arrhythmias, snoring, and sleep apnea. Chest.

[24] Stevenson IH, Teichtahl H, Cunnington D, Ciavarella S, Gordon I, Kalman JM (2008). Prevalence of sleep disordered breathing in paroxysmal and persistent atrial fibrillation patients with normal left ventricular function. Eur Heart J.

[25] Lopez-Jimenez F, Sert Kuniyoshi FH, Gami A, Somers VK (2008). Obstructive sleep apnea: Implications for cardiac and vascular disease: Part II: Contemporary reviews in sleep medicine. Chest.

[26] Koshino Y, Satoh M, Katayose Y, Yasuda K, Tanigawa T, Takeyasu N (2008). Association of sleep-disordered breathing and ventricular arrhythmias in patients without heart failure. Am J Cardiol.

[27] Punjabi NM, Newman A, Young T, Resnick HE, Sanders M (2008). Sleep disordered breathing and cardiovascular disease: An outcome-based definition of hypopneas. Am J Respir Crit Care Med.

[28] West SD, McBeath HA, Stradling JR (2009). Obstructive sleep apnoea in adults. BMJ.

[29] Tishler PV, Larkin EK, Schluchter MD, Redline S (2003). Incidence of sleep disordered breathing in an urban adult population: The relative importance of risk factors in the development of sleep-disordered breathing. JAMA.

[30] Kapur V, Strohl KP, Redline S, Iber C, O'Connor G, Nieto J (2002). Under diagnosis of sleep apnea syndrome in US communities. Sleep Breath.

[31] Young T, Peppard PE, Gottlieb DJ (2002). Epidemiology of obstructive sleep apnea: A population health perspective. Am J Respir Crit Care Med.

[32] Somers VK, White DP, Amin R, Abraham WT, Costa F, Culebras A (2008). Sleep Apnea and Cardiovascular Disease: An American Heart Association/American College of Cardiology Foundation Scientific Statement From the American Heart Association Council for High Blood Pressure Research Professional Education Committee, Council on Clinical Cardiology, Stroke Council, and Council on Cardiovascular Nursing In Collaboration With the National Heart, Lung, and Blood Institute National Center on Sleep Disorders Research (National Institutes of Health). Circulation.

[33] Randazo DN, Winters SL, Schweitzer P (1996). Obstructive sleep apnea induced supraventricular tachycardia. J Electrocardiol.

[34] Miller WP (1982). Cardiac arrhythmias and conduction disturbances in the sleep apnea syndrome: Prevalence and significance. Am J Med.

[35] Grimm W, Hoffmann J, Menz V, Kohler U, Heitmann J, Peter JH (1996). Electrophysiologic evaluation of sinus node function and atrioventricular conduction in patients with prolonged ventricular asystole during obstructive sleep apnea. Am J Cardiol.

[36] Zwillich C, Devlin T, White D, Douglas N, Weil J, Martin R (1982). Bradycardia during sleep apnea: Characteristics and mechanism. J Clin Invest.

[37] Fuster V, Ryden LE, Asinger RW, Cannom DS, Crijns HJ, Frye RL (2001). ACC/AHA/ESC guidelines for the management of patients with atrial fibrillation: Executive summary; A report of the American College of Cardiology/American Heart Association Task Force on practice guidelines and the European Society of Cardiology Committee for Practice Guidelines and Policy Conferences (Committee to Develop Guidelines for the Management of Patients With Atrial Fibrillation) developed in collaboration with the North American Society of Pacing and Electrophysiology. Circulation.

[38] Garvey JF, Taylor CT, McNicholas WT (2009). Cardiovascular disease in obstructive sleep apnea syndrome: The role of intermittent hypoxia and inflammation. Eur Respir J.

[39] Flemons WW, Remmers JE, Gillis AM (1993). Sleep apnea and cardiac arrhythmias: Is there a relationship?. Am Rev Respir Dis.

[40] Shepard JW, Garrison MW, Grither DA, Dolan GF (1985). Relationship of ventricular ectopy to oxyhemoglobin desaturation in patients with obstructive sleep apnea. Chest.

[41] Kanagala R, Murali NS, Friedman PA, Ammash NM, Gersh BJ, Ballman KV (2003). Obstructive sleep apnea and the recurrence of atrial fibrillation. Circulation.

[42] Harbison J, O'Reilly P, McNicholas WT (2000). Cardiac rhythm disturbances in the obstructive sleep apnea syndrome: Effects of nasal continuous positive airway pressure therapy. Chest.

[43] Veale D, Chailleux E, Hoorelbeke-Ramon A, Reybet-Degas O, Humeau-Chapuis MP, Alluin-Aigouy F (2000). Mortality of sleep apnoea patients treated by nasal continuous positive airway pressure registered in the ANTAVIR Observatory: Association Nationale pour le Traitement A Domicile de l'Insuffisance Respiratoire Chronique. Eur Respir J.

[44] Kelly A, Marcus CL (2005). Childhood obesity, inflammation, and apnea: What is the future for our children?. Am J Respir Crit Care Med.

[45] Tilkian AG, Guilleminault C, Schroeder JS, Lehrman KL, Simmons FB, Dement WC (1977). Sleep-induced apnea syndrome: Prevalence of cardiac arrhythmias and their reversal after tracheostomy. Am J Med.

[46] Becker H, Brandenburg U, Peter JH, von Wichert P (1995). Reversal of sinus arrest and atrioventricular conduction block in patients with sleep apnea during nasal continuous positive airway pressure. Am J Respir Crit Care Med.

[47] Mooe T, Gullsby S, Rabben T, Eriksson P (1996). Sleep-disordered breathing: A novel predictor of atrial fibrillation after coronary artery bypass surgery. Coron Artery Dis.

[48] Javaheri S, Parker TJ, Liming JD, Corbett WS, Nishiyama H, Wexler L (1998). Sleep apnea in 81 ambulatory male patients with stable heart failure: Types and their prevalences, consequences, and presentations. Circulation.

[49] Mitchell AR, Spurrell PA, Sulke N (2003). Circadian variation of arrhythmia onset patterns in patients with persistent atrial fibrillation. Am Heart J.

[50] Simantirakis EN, Schiza SI, Marketou E, Chrysostomakis SI, Chlouverakis GI, Klapsinos NC (2004). Severe bradyarrhythmias in patients with sleep apnoea: The effect of continuous positive airway pressure treatment A long-term evaluation using an insertable loop recorder. Eur Heart J.

[51] Gami AS, Pressman G, Caples SM, Kanagala R, Gard JJ, Davison DE (2004). Association of atrial fibrillation and obstructive sleep apnea. Circulation.

[52] Porthan KM, Melin JH, Kupila JT, Venho KK, Partinen MM (2004). Prevalence of sleep apnea syndrome in lone atrial fibrillation: A case-control study. Chest.

[53] Parati G, Di RM, Bonsignore MR, Insalaco G, Marrone O, Castiglioni P (1997). Autonomic cardiac regulation in obstructive sleep apnea syndrome: Evidence from spontaneous baroreflex analysis during sleep. J Hypertens.

[54] Narkiewicz K, Pesek CA, Kato M, Phillips BG, Davison DE, Somers VK (1998). Baro-reflex control of sympathetic nerve activity and heart rate in obstructive sleep apnea. Hypertension.

[55] de Paula PM, Tolstykh G, Mifflin S (2007). Chronic intermittent hypoxia alters NMDA and AMPA-evoked currents in NTS neurons receiving carotid body chemoreceptor inputs. Am J Physiol Regul Integr Comp Physiol.

[56] Narkiewicz K, van de Borne PJ, Montano N, Dyken ME, Phillips BG, Somers VK (1998). Contribution of tonic chemoreflex activation to sympathetic activity and blood pressure in patients with obstructive sleep apnea. Circulation.

[57] Allesie M, Ausma J, Schotten U (2002). Electrical, contractile and structural remodeling during atrial fibrillation. Cardiovasc Res.

[58] Ariyarajah V, Spodick DH (2006). The Bachmann bundle and inter-atrial conduction. Cardiol Rev.

[59] Wit AL, Rosen AR, Fossard HA, Haber E, Jenning RB, Katz M, Morgan E (1986). After depolarization and triggered activity. The heart and cardiovascular system.

[60] Nieto FJ, Young TB, Lind BK, Shahar E, Samet JM, Redline S (2000). Association of sleep-disordered breathing, sleep apnea, and hypertension in a large community-based study. JAMA.

[61] Peppard PE, Young T, Palta M, Skatrud J (2000). Prospective study of the association between sleep-disordered breathing and hypertension. N Engl J Med.

[62] Kannel WB, Wolf PA, Benjamin EJ (1998). Prevalence, incidence, prognosis, and predisposing conditions for atrial fibrillation: Population-based estimates. Am J Cardiol.

[63] Chugh SS, Blackshear JL, Shen WK, Hammill SC, Gersh BJ (2001). Epidemiology and natural history of atrial fibrillation: Clinical implications. J Am Coll Cardiol.

[64] Baranchuk A, Simpson CS, Redfearn DP, Fitzpatrick M (2008). It's time to wake up! Sleep Apnea and Cardiac Arrhythmias. Europace.

[65] Condos WR, Latham RD, Hoadley SD, Pasipoularides A (1987). Hemodynamics of the Mueller maneuver in man: Right and left heart micromanometry and Doppler echocardiography. Circulation.

[66] Hall MJ, Ando S, Floras JS, Bradley TD (1998). Magnitude and time course of hemodynamic responses to Mueller maneuvers in patients with congestive heart failure. J Appl Physiol.

[67] Franz MR, Bode F (2003). Mechano-electrical feedback underlying arrhythmias: The atrial fibrillation case. Prog Biophys Mol Biol.

[68] Shamsuzzaman AS, Winnicki M, Lanfranchi P, Wolk R, Kara T, Accurso V (2002). Elevated C-reactive protein in patients with obstructive sleep apnea. Circulation.

[69] Chung MK, Martin DO, Sprecher D, Wazni O, Kanderian A, Carnes CA (2001). C-reactive protein elevation in patients with atrial arrhythmias: Inflammatory mechanisms and persistence of atrial fibrillation. Circulation.

[70] Otto ME, Belohlavek M, Romero-Corral A, Gami AS, Gilman G, Svatikova A (2007). Comparison of cardiac structural and functional changes in obese otherwise healthy adults with versus without obstructive sleep apnea. Am J Cardiol.

[71] Romero-Corral A, Somers VK, Pellikka PA, Olson EJ, Bailey KR, Korinek J (2007). Decreased right and left ventricular myocardial performance in obstructive sleep apnea. Chest.

[72] Madden BP, Shenoy V, Dalrymple-Hay M, Griffiths T, Millard J, Backhouse L (1997). Absence of bradycardic response to apnea and hypoxia in heart transplant recipients with obstructive sleep apnea. J Heart Lung Transplant.

[73] Daly MD, Angell-James JE, Elsner R (1979). Role of carotid-body chemoreceptors and their reflex interactions in bradycardia and cardiac arrest. Lancet.

[74] Somers VK, Dyken ME, Mark AL, Abboud FM (1992). Parasympathetic hyperresponsiveness and bradyarrhythmias during apnoea in hypertension. Clin Auton Res.

[75] Litvin AY, Pevzner AV, Golitsyn PV, Galyavi RA, Mazygula EP, Nesterenko LY (2006). New approaches to treatment of bradyarrhythmia in patients with obstructive sleep apnea syndrome. Terapevtičskij Arhiv.

[76] Koehler U, Fus E, Grimm W, Pankow W, Schafer H, Stammnitz A (1998). Heart block in patients with obstructive sleep apnoea: Pathogenetic factors and effects of treatment. Eur Respir J.

[77] Han J, Millet D, Chizzonitti B, Moe GK (1966). Temporal dispersion of recovery of excitability in atrium and ventricle as a function of heart rate. Am Heart J.

[78] Conti JB (2005). Cardiac arrhythmias. J Am Coll Cardiol.

[79] Franz MR (1996). Mechano-electrical feedback in ventricular myocardium. Cardiovasc Res.

[80] Stegman SS, Burroughs JM, Henthorn RW (1996). Asymptomatic bradyarrhythmias as a marker for sleep apnea: Appropriate recognition and treatment may reduce the need for pacemaker therapy. Pacing Clin Electrophysiol.

[81] Ryan CM, Usui K, Floras JS, Bradley TD (2005). Effect of continuous positive airway pressure on ventricular ectopy in heart failure patients with obstructive sleep apnoea. Thorax.

[82] Marin JM, Carrizo SJ, Vicente E, Agusti AG (2005). Long-term cardiovascular outcomes in men with obstructive sleep apnoea-hypopnoea with or without treatment with continuous positive airway pressure: An observational study. Lancet.

[83] Garrigue S, Bordier P, Jaäs P, Shah DC, Hocini M, Raherison C (2002). Benefit of atrial pacing in sleep apnea syndrome. N Engl J Med.

[84] Lüthje L, Unterberg-Buchwald C, Dajani D, Vollman D, Hasenfuss G, Andreas S (2005). Atrial overdrive pacing in patients with sleep apnea with implanted pacemaker. Am J Respir Crit Care Med.

[85] Pépin JL, Defaye P, Garrigue S, Poezevara Y, Lévy P (2005). Overdrive pacing does not improve obstructive sleep apneoa syndrome. Eur Respir J.

[86] Simantirakis EN, Schiza SE, Chrysostomakis SI, Chlouverakis GI, Kalapsinos NC, Siafakas NM (2005). Atrial overdrive pacing for the obstructive sleep apnea-hypopnea syndrome. N Engl J Med.

[87] Krahn AD, Yee R, Erickson MK, Markowitz T, Gula LJ, Klein GJ (2006). Physiologic pacing in patients with obstructive sleep apnea: A prospective, randomized crossover trial. J Am Coll Cardiol.

[88] Sharafkhaneh A, Sharafkhaneh H, Bredikus A, Guilleminault C, Bozkurt B, Hirshkowitz M (2007). Effect of atrial overdrive pacing on obstructive sleep apnea in patients with systolic heart failure. Sleep Med.

[89] Punjabi NM, Welch D, Strohl K (2000). Sleep disorders in regional sleep centers: A national cooperative study: Coleman II Study Investigators. Sleep.

[90] Baranchuk A, Simpson CS, Redfearn DP, Michael K, Fitzpatrick M (2008). Understanding the association between sleep apnea and cardiac arrhythmias. REA.

